# Role of Liberal Primary Fasciotomy in Traumatic Vascular Injury

**DOI:** 10.5812/traumamon.5368

**Published:** 2012-07-31

**Authors:** Farooq Ahmad Ganie, Hafeezulla Lone, Mohd Lateef Wani, Farooq Ahmad Dar, Nasir-u-din Wani, Shadab Nabi Wani

**Affiliations:** 1 Department of Resident Cardiovascular and thoracic surgery, SKIMS Soura, Srinagar, India

**Keywords:** Primary Liberal Fasciotomy, Compartment Syndrome, Revision Fasciotomy, Delayed Fasciotomy

## Abstract

**Background:**

Vascular injury represents less than 1% of all injuries, but deserves special attention because of its severe complications. Amputation or retention of a painful functionless limb is the most untoward result of severe vascular injury or inadequate treatmet. Thus, vascular injury needs a judicious and multidimensional approach.

**Objectives:**

This retrospective study was done to asess the outcome of minor modifications of the methodology of extremity fasciotomy by making it liberal with respect to incision and definition.

**Materials and Methods:**

Out of 55 patients in 2008, 45 patients (Group A) had either no fasciotomy or limited primary fasciotomy, 10 patients (Group B) had primary liberal fasciotomy. Another group from 2008 onwards had undergone primary liberal fasciotomy in all the 45 patients (Group C).

**Results:**

In group A, we had 5 amputations and one death. In group B, there were no amputations or deaths and from group C, we had one amputation and no deaths.

**Conclusions:**

Blunt and distal traumatic vascular injury of the extremities and its repair should always combined with primary liberal fasciotomy, which although increases manageable morbidity, avoids disability (functional as well as anatomical).

## 1. Background

Vascular injury represents less than 1% of all injuries, but deserves special attention because of their severe complications. Amputation or retention of a painful functionless limb is the most untoward result of severe vascular injury or inadequate treatment, so vascular injury needs a judicious and multidimensional approach. Patients with pain out of proportion to injury, pain upon passive stretching, sensory changes, weakness or parasthesia after vascular repair indicate vascular compromise due to compartment syndrome and need immediate fasciotomy. Popliteal artery injuries continue to result in maximum limb loss, possibility due to use of limited fasciotomy.

## 2. Objectives

This study was done to assess the outcome of minor modifications of the methodology of extremity fasciotomy by making it liberal with respect to incision and definition.

## 3. Materials and Methods

### 3.1. Study Population

We studied 55 patients in 2008 with firearm or splinter vascular injuries of extremities; 45 patients (Group A) underwent different methods of vascular repair either without primary fasciotomy or with limited fasciotomy. Only 10 patients (Group B) had liberal primary fasciotomy. After 2008 to date, we treated 45 patients (Group C) with different types of vascular injuries, by different reparatory methods all of which received primary liberal fasciotomy.

### 3.2. Pre-operative Evaluation

Assessment included emergency work-up: clinical examination, CBC, KFT, ECG, chest x-ray, USG abdomen, color flow Doppler, blood grouping and cross-matching; only 6 patients were subjected to pre-operative angiography.

### 3.3. Surgical Procedure

Vascular repair was done by primary end-to-end anastomosis or reverse sephanous vein graft either without fasciotomy or with varied limits of fasciotomy.

### 3.4. Fasciotomy

Limited fasciotomy included superficial incision with inadequately cut deep fascia, isolated compartment fasciotomy, inadequate length of fasciotomy not passing across proximal and distal joints. Liberal fasciotomy included cutting through skin, deep fascia as well as outer covering of underlying exposed muscles (epimysium), till muscle pouts out. Oozing blood from muscle indicates adequate blood flow through the repaired vessel as well as adequacy of fasciotomy, thus it has therapeutic as well as diagnostic importance. Checking muscle viability with low voltage electric cautery stimulation. Confirming muscle viability helps in adequate debridement to prevent infective complications of dead tissue.

### 3.5. Type of Incision

We use S-shaped incision both at elbow and popliteal fossa, closure of this incision does not cause any constricting effect, while in case of liberal primary fasciotomy, the same incision is extended. The repaired vessel is loosely covered either by surrounding fat or muscle to prevent desiccation of the vessel. Curved fasciotomy incisions decompress the maximum area of the extremity and avoids superficial venous injury.Ensure muscle pouting along fasciotomy wound. All-compartmental fasciotomy is better than isolated-compartment fasciotomy. Dressings should not be tight. Avoid entrapment of adventitia in the anastomotic suture line.

Passing a Fogarty catheter damages endothelium and increases tendency of thrombosis, thus anticoagulation is recommended.

## 4. Results 

From group A, we had 5 amputations and one death (death due to infective complications of gangrene followed by DIC - despite that the limb was amputated); from group B we had no complications and from group C one amputation and no deaths were recorded; 12 patients from group A needed either extension of fasciotomy or secondary fasciotomy.

Most of the patients with liberal primary fasciotomy need care by a plastic surgeon. But 25% patients were discharged and referred to their respective primary health care centers for regular dressings and admitted subsequently for split-thickness skin grafting with favorable results.

All those patients with primary liberal fasciotomy even with borderline muscle viability at the time of primary vascular repair had the least amputation rate. There was no significant increase in infection rate. Soaked dressings were changed regularly. One significant complication with primary liberal fasciotomy was pain which needed short interval analgesics. Also changing dressings in these patients was time consuming and significantly painful which demanded extra patience by patient as well as attending resident. These patients have long lasting paresthesia at graft site with varied presentation. Patients in high dependency units with no or limited fasciotomy obscured signs of compartment syndrome due to liberal use of analgesics. Delayed or revised fasciotomy in these patients helped to a very limited extent, and in the long run gave a functionally disabled limb with chronic pain in saved limbs.

### 4.1. Psychological Impact

Delayed fasciotomy, revision fasciotomy and disability due to amputation/vegetative limb or chronic limb parasthesia all have a very strong psychological impact in contrast to less morbidity associated with primary liberal fasciotomy.

In patients with primary liberal fasiotomy crossing knee and ankle in lower limb, elbow and wrist in upper limb with cutting some fibers of reticulum at ankle or wrist appreciably improved blood flow. Any vascular injury associated with blunt trauma limb, fracture, venous injury, longer duration of ischemia; below knee/elbow vascular repair needed primary liberal fasciotomy whether the patient had a tense compartment at the time of vascular repair or not.

### 4.2. In Hospital Morbidity

Extensive soakage from fasciotomy wound needs frequent change of dressings, which is painful and cumbersome for patient. Frequent analgesics make patients apprehensive. Painful postural changes and difficulty in assuming comfortable postures effects sleep. Longer hospital stay is uncomfortable.

## 5. Discussion

Compartment syndrome is a surgical emergency characterized by raised pressure in an unyielding osteofascial compartment caused by trauma, revascularization, myocyte edema after ischemia-reperfusion injury, or resuscitation (1-4). The common sites of compartment syndrome are the lower legs (53%–62%), followed by forearm (24%–26%), thigh (4%–15%), foot (4%–5%), and hand (5). A close association exists between grade of fracture, degree of combination, and risk of developing compartment syndrome in open tibial fractures (6). Our experience concluded that firearm and crush injuries, fractures, massive transfusions with coagulopathy, contaminated wounds and local sepsis increase the risk of compartment syndrome. The most common revision procedures were extension of fascial incisions and opening a new compartment particularly the anterior compartment of the lower leg (7). Delayed fasciotomy has twice the rate of amputation and threefold higher mortality (7). We noted that delayed or revised fasciotomy increase morbidity, mortality as well as disability compared to the manageable morbidity of primary liberal fasciotomy. Internal fixation of fracture in the presence of open fasciotomy wound does not increase infection risk (8). Compartment syndrome despite fasciotomy increases amputation rate significantly due to limited primary fasciotomy (9, 10). We observed that delayed or revised fasciotomy decreases confidence of the patient and leads to severe psychological disturbance negatively effecting patient’s co-operation. Anticoagulant treatment should be given to prevent thrombosis due to endothelial trauma by Fogarty catheter. Early fasciotomy is warranted if there is any suspicion of occurrence of compartment syndrome (11). Subcutaneous fasciotomy does not always ensure sufficient decompression of all four lower leg compartments (12). Complications related to fasciotomy are rare (12). When revascularization is made after six hours, the prophylactic fasciotomy is recommended (13). Early fasciotomy may reduce amputation rates in extremity arterial injury (14). Popliteal artery trauma results in amputation more often than any other arterial injury (15). Primary liberal fasciotomy crossing knee and ankle increases healing rate of the wound compared to delayed liberal fasciotomy. Four-compartment fasciotomies are required to restore and preserve adequate distal flow (16). Voilent forces, associated muscle and integument trauma, longer pre-operative and operative warm ischemia time, all favour tissue edema that may progress to compartment syndrome and require immediate decompressing fasciotomy (17). Liberal fasciotomy is not a defined term, but needs to be defined with respect to the primary operative finding and there is no borderline when and when not to perform liberal fasciotomy. In view of irreversible neurologic and muscular sequelae of a missed compartment syndrome, there has been a trend for a prophylactic fasciotomy in peripheral vascular injuries(18). We conclude that fasciotomy can never be called as prophylactic; rather it has a pivotal role in primary setting of vascular repair. Successful management correlates best with prompt repair of both popliteal arterial and venous injuries with early fasciotomy (19). The smaller the vessel repaired, the more is the need of liberal fasciotomy. Liberal fasciotomy wounds can be closed primarily or by split skin graft 10 to 15 days later. We studied no significant increase in use of hospital resources in case of liberal primary fasciotomy.

Blunt and distal traumatic vascular injury of extremities and its repair should be combined with primary liberal fasciotomy, which may although increase manageable morbidity, will avoid lifelong disability. No fasciotomy can be acceptable when chances of compartment syndrome are absolutely nil; however, limited fasciotomy is absolutely discouraged in favor of primary liberal fasciotomy.
